# Numerical analysis of the ostiomeatal complex aeration using the CFD method

**DOI:** 10.1038/s41598-023-31166-x

**Published:** 2023-03-09

**Authors:** Dmitry Tretiakow, Krzysztof Tesch, Karolina Markiet, Tomasz Przewoźny, Aida Kusiak, Dominika Cichońska, Andrzej Skorek

**Affiliations:** 1 Department of Otolaryngology, Medical University of Gdansk, Gdańsk, Poland; 2 Faculty of Mechanical Engineering and Ship Technology, Gdansk University of Technology, Gdańsk, Poland; 3 II Department of Radiology, Medical University of Gdansk, Gdańsk, Poland; 4 Department of Periodontology and Oral Mucosa Diseases, Medical University of Gdansk, Gdańsk, Poland

**Keywords:** Computational models, Computational biology and bioinformatics, Anatomy, Diseases, Risk factors, Signs and symptoms

## Abstract

We aimed to analyse ostiomeatal complex (OMC) aeration using the computational fluid dynamics (CFD) method of simulation based on human craniofacial computed tomography (CT) scans. The analysis was based on CT images of 2 patients: one with normal nose anatomy and one with nasal septal deviation (NSD). The Reynolds-Average Simulation approach and turbulence model based on linear eddy viscosity supplemented with the two-equation *k*-$\omega$ SST model were used for the CFD simulation. As a result, we found differences in airflow velocity through the ostiomeatal complex in patients with a normal nose and those with NSD. In a patient with NSD, the flow is turbulent in contrast to the normal nose (laminar flow). A faster (more intensive) airflow through the OMC was observed in the wider nasal cavity of the patient with NSD than on the narrower side. In addition, we want to emphasise the higher speed of airflow through the apex uncinate process area towards the ostiomeatal complex during exhalation, which, in the presence of secretions in the nose, predisposes to its easier penetration into the sinuses of the anterior group.

## Introduction

**Figure 1:**
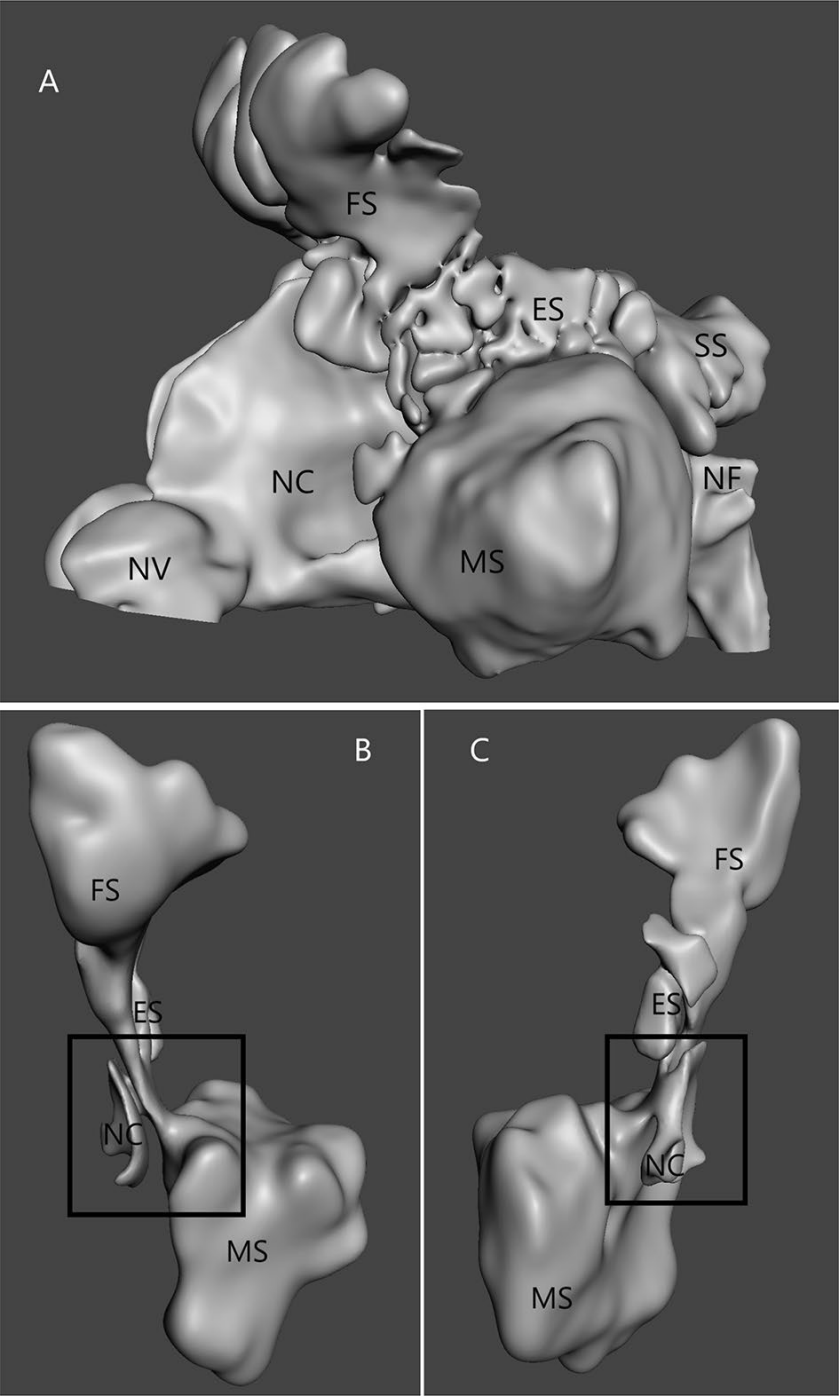
(**A**) 3D model of the nasal cavity (NC) and nasal sinus (ES—ethmoid sinus; MS—maxillar sinus; FS—frontal sinus; SS—sphenoid sinus; NV—nasal vestibule); (**B**) isolated the left ostiomeatal complex (black quad) region, anterior view; (**C**) isolated the left ostiomeatal complex (black square) region, posteromedial view.

The ostiomeatal complex (OMC) is the anatomical structure that connects the frontal, maxillary and ethmoid sinuses with the nasal cavity (Fig. 1). Most of the inflammatory processes in this group of sinuses simultaneously originate in the nasal cavity and spread to one or more sinuses (sinusitis/pansinusitis)^1^. Pathologies in the OMC are both the cause and the result of inflammatory processes in these sinuses (the typical vicious cycle). We can talk about three aspects of the OMC pathology:

1. impaired patency associated with congenital/developmental or acquired structural changes,
2. dysfunction of the mucociliary transport from the sinus to the nasal cavity, and
3. the overlooked topic of airflow through this part of the nose.

Congenital/developmental OMC obstruction may be related to anatomical changes such as concha bullosa, lowering of the orbital floor, or the presence of Haller cells or agger nasi cells, the depth of the olfactory groove. The patency of the OMC may also be conditioned by variants of the anatomical structure, namely the type of uncinate process setting^2^ or the length of the cribriform plate^3,4^. Furthermore, acquired OMC obstruction may be associated with a foreign body, an inflammatory, neoplastic or traumatic process. Besides the patency of the OMC, when assessing the physiology of the mucociliary transport, the condition of the epithelium and the physical/chemical properties of the mucus covering it should also be taken into account (gel phase vs. liquid-sol phase). It is also worth paying attention to the general biocenotic and biochemical factors occurring at the nasal/sinus border: bacteriological differences, the concentration of nitric oxide, concentration of substances that slow down/immobilise the cilia, air temperatures and humidity, changes of the pH^5–8^. All these elements can quantitatively and qualitatively affect the efficiency of the OMC (also known as ‘nasal patency’)^9^. Our study directly concerns the issue of airflow within the ostiomeatal complex during the various phases of the respiratory cycle in patients with normal anatomy and nasal septum deviation. The aim of this study was to measure if nasal septum deviation alters the airflow through the OMC.

## Results

**Figure 2:**
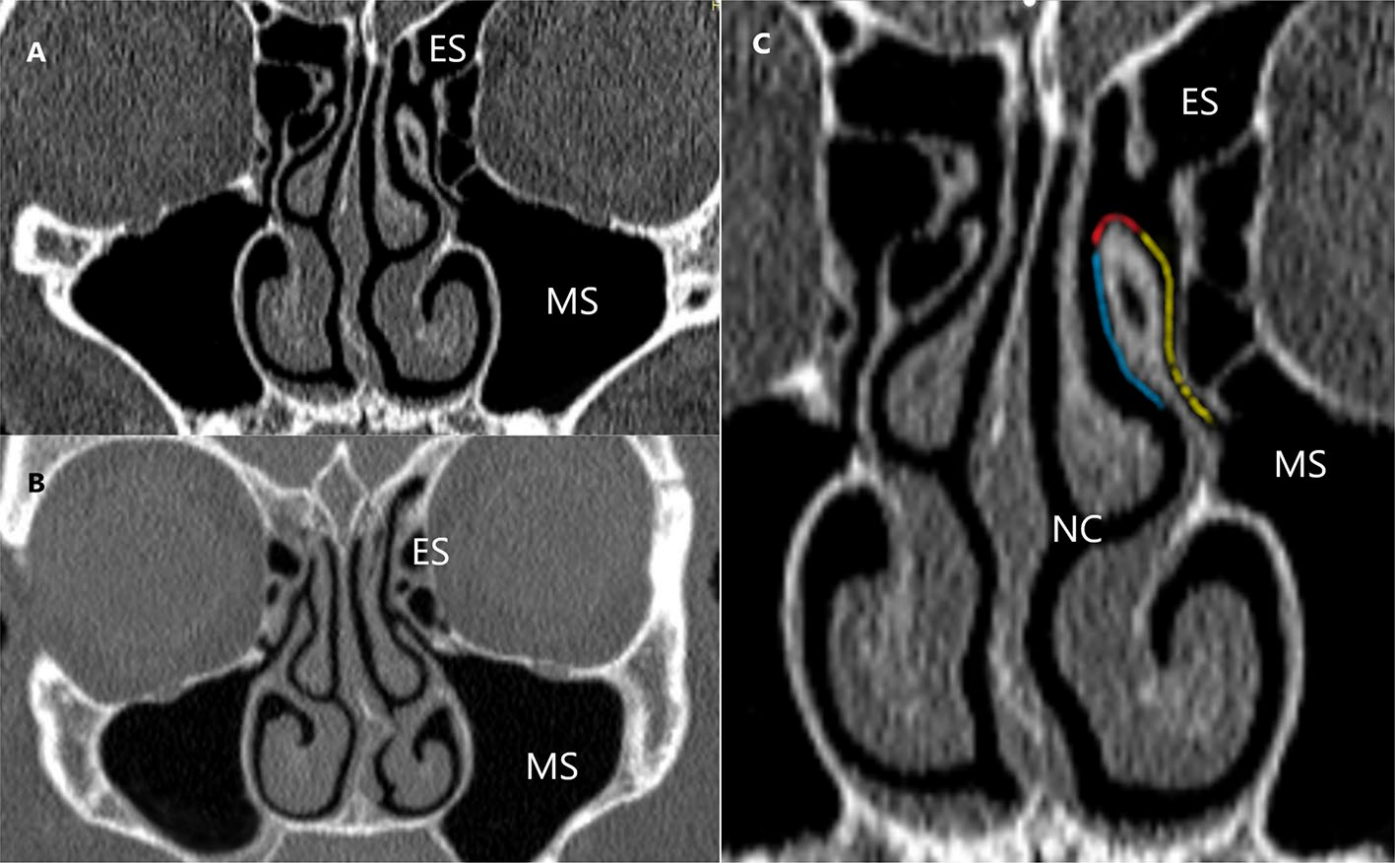
CT scans of nasal sinuses (ES—ethmoid sinus; MS—maxillar sinus; NC—nasal cavity): (**A**) Patient 1 (normal anatomy); (**B**) Patient 2 with nasal obstruction and nasal septum deviation (NSD) to the left side; (**C**) sinus surface of the ethmoid bone uncinate process (SUP) (yellow), the nasal surface of the ethmoid bone uncinate process (NUP) (blue); apex of the ethmoid bone uncinate process (AUP) (red).

The CT scans of Patient 1 were used to create the computational model of the normal nasal cavity and nasal sinuses. Patient 2 had nasal septum deviation (NSD) causing nasal obstruction but without pathological changes in the maxillary sinuses. We assume the direction of airflow along the sinus (SUP) and nasal (NUP) surfaces of the uncinate process of the ethmoid bone and at its highest point (AUP) (Fig. 2).

### Patient 1: inspiration phase (Fig. 3)

During the initial period of inspiration, the air at SUP, AUP, and NUP areas moved towards the posterior nostrils. However, while in the NUP area, the air moved backwards in a laminar manner. In the upper section of the SUP area we observed the flow towards the AUP point and further to the posterior nostrils and in the lower section into the interior of the maxillary sinus. There were no significant differences in airflow velocities. In the middle and late periods of the inspiration phase the airflow direction did not change, however we observed significant differences in speed and volume. The flow through the NUP was 4 $\times$ greater than through the SUP (0.4 $\text {m}\,\text {s}^{-1}$ vs. 0.1 $\text {m}\,\text {s}^{-1}$). In the AUP, we observed the flow direction towards the SUP and the choanal nares. The flow rate was variable and decreased beyond the AUP. In addition, at each stage of inhalation, at the base of the SUP, we observed airflow into the maxillary sinus.

**Figure 3:**
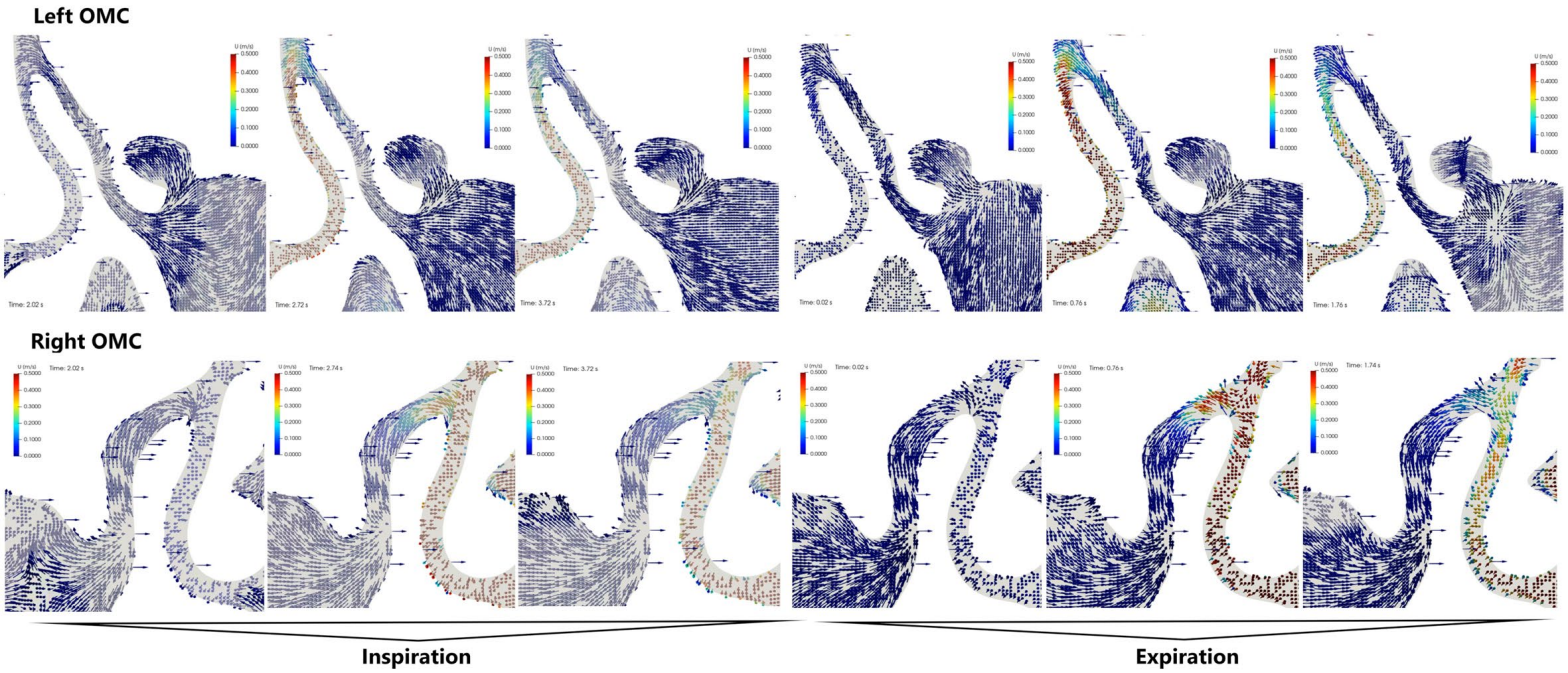
Bilateral visualization of the airflow in the ostiomeatal complex (frontal projection) of Patient 1. The inspiration and expiration phases were 2 s for each. For each phase, the results were presented in 3 sequences: the beginning of the phase (first 0.2–0.3 s), half point (0.9–1.0 s), and the end (1.8–2.0 s). Measurements were obtained at the start of each phase.

### Patient 1: expiration phase (Fig. 3)

In the initial period of exhalation, the air flowed through the NUP and the SUP areas towards the anterior nostrils. No differences in air flow velocities were observed. However, in the middle period of the expiration phase, we observed a 4-fold difference in flow velocity (NUP > SUP) 0.4  $\text {m}\,\text {s}^{-1}$ vs. 0.1 $\text {m}\,\text {s}^{-1}$). This was because the flow through the NUP and SUP areas during expiration was laminar. Therefore, in the AUP point, as in the inspiration phase, we observed a decrease in the speed and direction of flow after crossing the highest point, while in the part closer to the NUP area, the flow was forward, in part closer to the SUP the direction is towards the inside of the OMC.

When analysing the flows through the OMC in Patient 1 (inhalation and exhalation phase), it can be determined that:

1. During the middle part of both breathing phases, there is a significant (4 $\times$) difference in the flow rate between the NUP and SUP areas.
2. At the AUP point, we observe a gradual decrease in the airflow velocity along with the movement towards the OMC.
3. In the NUP and SUP areas the airflow is laminar.
4. Air enters the maxillary sinus during all phases of breathing.

### Patient 2: inspiration phase (Fig. 4)

The presented image of flows concerns a patient with left-sided nasal septal deviation. During inhalation, we observed a different flow of air through the nasal cavity and OMC depending on which side the nasal septum deviated.

On the right (wider) side, during the initial period of inspiration, airflow was observed in the NUP area towards the posterior nostrils and in the SUP area—towards the anterior nostrils and the maxillary sinus. In the middle period of the inspiration phase, the airflow through the NUP and SUP areas was posterior, with slight differences in flow velocity (volume) (0.5 vs. 0.4 $\text {m}\,\text {s}^{-1}$—ratio 1.25). The fastest flow occurred in the SUP middle section, while in the AUP, the flow velocity was minimal but towards the OMC. In the final period of inspiration, the difference in speed was significant (5 $\times$) in favour of NUP (> SUP) (0.5 $\text {m}\,\text {s}^{-1}$ vs. 0.1 $\text {m}\,\text {s}^{-1}$).

On the left (narrower) side, in the initial period of inspiration, the airflow in the area of the NUP was towards the posterior nares and in the area of the SUP towards the anterior nostrils and the maxillary sinus. There was no difference in airflow velocity. In the middle period of inspiration, there was a clear difference in flow velocities around NUP and SUP—0.5 $\text {m}\,\text {s}^{-1}$ vs. 0.1 $\text {m}\,\text {s}^{-1}$ (ratio 5$\times$). We observed a slightly lower flow at the AUP point than at the NUP area (0.3 $\text {m}\,\text {s}^{-1}$) posteriorly and towards the maxillary sinus. In the final period of the inspiration phase, the flow was similar to its middle part. During inspiration, the airflow was not laminar, as we observed differences in the flow velocity between the layers of air around the NUP.

**Figure 4:**
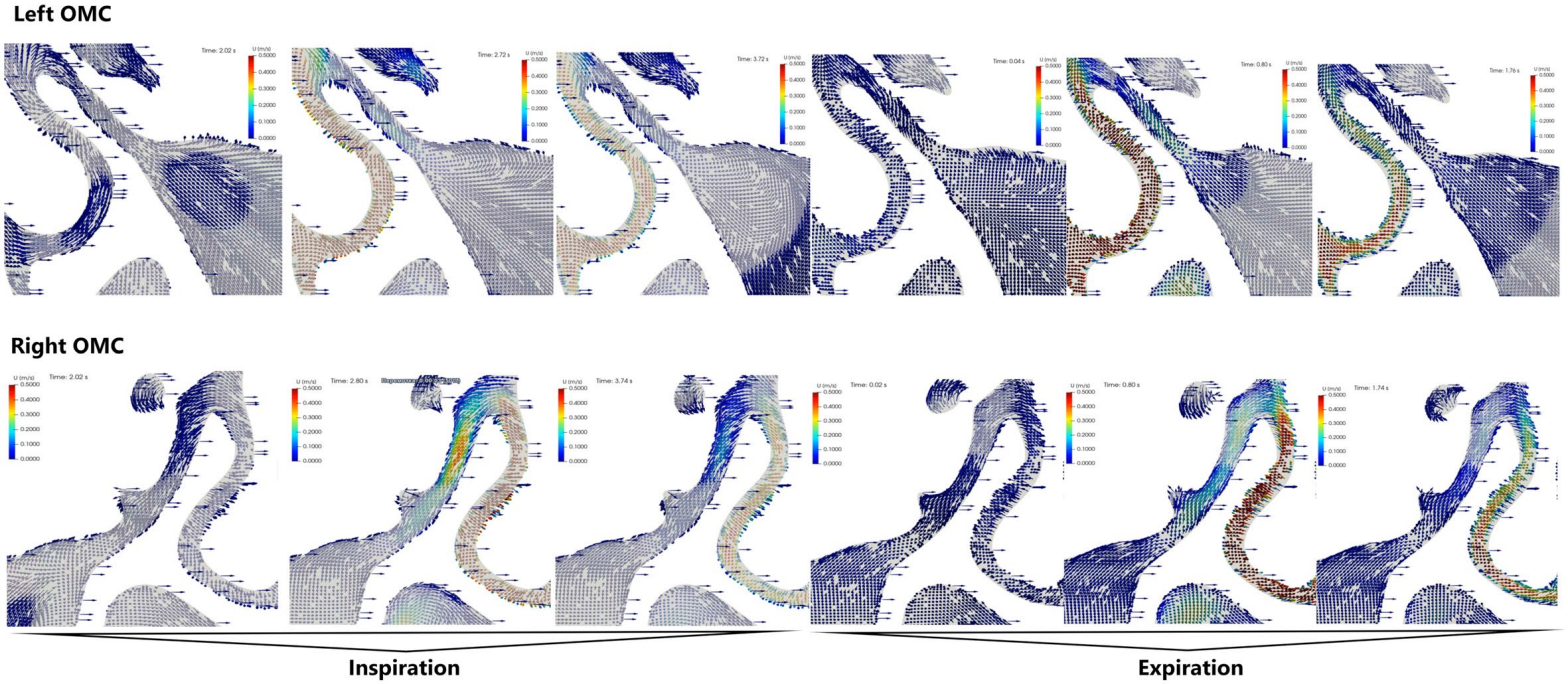
Bilateral visualization of the airflow in the ostiomeatal complex (frontal projection) of Patient 2. The inspiration and expiration phases each had duration of 2 s. For each phase, the results were presented in 3 sequences: the beginning of the phase (first 0.2–0.3 s), half point (0.9–1.0 s) and the end (1.8–2.0 s). Measurements were obtained at the start of each phase.

### Patient 2: expiration phase (Fig. 4)

On the right (wider) side, in the initial period of expiration, the air in the NUP and SUP areas moved towards the anterior nostrils and from the maxillary sinus without differences in flow rates. In the middle phase of expiration, the airflow velocity increased (NUP/SUP—0.5 $\text {m}\,\text {s}^{-1}$ vs. 0.3 $\text {m}\,\text {s}^{-1}$ ratio 1.67). Around the AUP, the flow velocity was similar to the flow through the OMC. The flow around NUP was not laminar. Differences in flow velocities in the individual layers and flow directions were observed. In the final phase of expiration, the difference in flow velocities decreases (NUP vs. SUP—0.3 $\text {m}\,\text {s}^{-1}$ vs. 0.1 $\text {m}\,\text {s}^{-1}$). The airflow direction in the NUP area was towards the anterior nostrils, while in the SUP and OMC, the flow direction was away from the maxillary sinus.

On the left (narrower) side, in the initial period of expiration, the airflow velocity in the area of SUP, NUP and AUP areas was comparable and directed towards the anterior nostrils and from the maxillary sinus. In the expiratory phase’s middle period, the flow velocity difference around NUP vs. SUP was 0.5 $\text {m}\,\text {s}^{-1}$ vs. 0.2 $\text {m}\,\text {s}^{-1}$ (ratio 2.5). The airflow is non-laminar (especially in proximity to the AUP) and the flow direction was towards the anterior nostrils and away from the maxillary sinus. In the final section of the exhalation phase, the flow properties were similar to its middle section. In addition, a significant difference in the flow velocity through the nasal cavity was observed. In the AUP, the flow velocity (as in the SUP) was 0.1 $\text {m}\,\text {s}^{-1}$ and increased to 0.5 $\text {m}\,\text {s}^{-1}$ closer to the base of the NUP. The airflow was non-laminar.

When analysing the airflow through the nose in a patient with septal deviation (inspiration and expiration phases), it can be determined that:

1. During the middle period of both respiratory phases, there was a difference in flow velocity between the NUP and SUP areas (1.25; 1.67) concerning the wider nasal cavity side. This difference to the narrower side is 5 and 2.5, respectively.
2. From the AUP point, we observe an increase in the airflow velocity toward the OMC on the ‘wider’ side and a decrease on the ‘narrower’ side.
3. The airflow was not laminar in the NUP and SUP areas.
4. Air entered the maxillary sinus during all phases of respiration.

## Discussion

Defence mechanisms protecting against the development of inflammatory processes in the nose and paranasal sinuses have an anatomical dimension (including the structure of the nasal vestibule, nasal valves and nasal turbinates), histological (specific type of epithelium and its structure) and molecular^10–12^. There is little research on the problem of airflow through the nasal cavity in the area of the lateral wall of the nose and the lumen of the paranasal sinuses and its possible impact on the development of inflammation. On one hand, pathogenic microbes and environmental pollutants enter the nose with the inhaled air. On the other, the mechanism of airflow through the nose and sinuses and its effect on the mucosa are overlooked in the pathophysiology of inflammation. Mucociliary transport is known to be directed towards the maxillary sinus ostea and further to the nasopharynx^1,13,14^. At the same time, the role of the airflow direction remains unclear: does it interact with this transport or is it opposite it? Is the air moving through the nasal cavity towards the lower part of the respiratory tract (during inspiration) purified on the surface of the nasal walls by a counter-current mechanism? In a sense, the answers to these questions is the results of our research. The airflow through the nose (particularly in the case of pathologies such as nasal septum deviation) is not laminar. The closer to the surface of the nasal mucosa, the faster the airflow and changing direction. The natural consequence of these differences is that a larger volume of air flows along the walls and ‘hits’ the epithelium from different sides. More significant contact of a larger volume of air with the epithelium, combined with the unique structure of the nasal turbinates, affects its temperature, humidity, and the possibility of purification. The greater force of air impact can lead to remodelling of the epithelium in different directions:

1. less patent nasal passage due to the proximity of the walls may block the free mucociliary transport, and
2. a higher viscosity of the mucus was observed, which may lead to a greater susceptibility of the nasal mucosa to infection^10,15^.

The mechanism of non-laminar airflow through the nose seems to work together with the mechanisms of changing the volume of the turbinates (and thus changing the surface and properties of the epithelium) to improve the quality of inhaled air.

Comparison of airflow in patients with normal nose and NSD reveals a change in airflow velocity (and thus its volume) through the spaces of the nasal cavity and the OMC (assessed in the area of SUP and NUP). In addition, on the side of the wider nasal cavity, we observe faster (more intensive) airflow through the OMC than on the less patent side (Fig. 4).

Doo et al. found correlations between the development of fungal ball sinusitis and NSD^16^. Other authors found correlations between the development of chronic sinusitis and anatomical anomalies in the nose: concha bullosa, ager nasi cells, Haller cells and NSD^4,10–12,17,18^. Atsal et al. noted that the frequency of these nasal anomalies increases with an increasing angle of deviation of the nasal septum from the midline^19^. Although on the other hand, there are opinions that since NSD affects 44-80% of the population, it is more accurate to talk about the coexistence of NSD with chronic rhinitis^20–22^. However, all authors agree that when NSD is accompanied by or results in changes in the OMC, this leads to the development of the inflammatory process of the nasal sinuses. Our research points to a potential reason for this—different airflow through the OMC. In addition, we want to emphasise the higher velocity of airflow through the AUP towards the OMC during expiration, which, in the presence of secretion in the nose, predisposes it to its easier penetration into the anterior group of the paranasal sinuses.

The issue of the extent of septoplasty in NSD remains open. Based on our research, we propose individualising this procedure and supplementing it with endoscopic uncinectomy when an OMC anomaly accompanies the NSD. Therefore, more research is needed. Future studies on large groups of patients may confirm or reject our hypothesis and explain the ranges of measurements of airflow turbulence within the ostiomeatal complex in healthy patients and those with nasal cavity pathology.

## Conclusions

CFD analysis of the ostiomeatal complex based on CT scans of the nasal sinuses allows for the simulation of airflow and its quantification analysis in patients with and without nasal cavity pathology. A comparison of airflow in patients with normal nose and NSD revealed a change in airflow velocity through the OMC . In addition, a faster (higher) airflow through the OMC was observed on the wider side than on the less permeable side. According to the literature, the NSD is accompanied by changes in the OMC, which leads to the development of the inflammatory process of the nasal sinuses. Our research points to a potential reason for this: different airflow through the OMC . In addition, we want to emphasise the higher speed of airflow through the AUP towards the OMC during exhalation. In the presence of secretions in the nose this predisposes to its easier penetration into the sinuses of the anterior group.

## Materials and Methods

### Nasal sinuses model

The computed tomography (CT) scans of the head and nasal sinuses were obtained from a patient without ENT pathology (Patient 1) and patient reporting to the Otolaryngology Outpatient Department due to difficulties with nasal breathing (Patient 2). The CT images (Fig. 2) were obtained in axial planes with multiplanar reconstructions with a slice thickness of 0.6–0.75 mm, resolution of 512 $\times$ 512 pixels, and pixel size of 0.3906 $\times$ 0.3906 mm. 3-D Slicer and Autodesk®Meshmixer (Autodesk Inc., San Francisco, USA) programs were used for image processing and model rendering (Fig. 1). A detailed description of the model preparation process was described in our previous publication^9^. The evaluation of the flow studies was performed separately for inspiration and expiration. This study focused on assessing air movement in the ostiomeatal complex region and was conducted by two experienced otolaryngologists, who also interpreted the results independently.

The Regional Bioethics Committee of the Medical University of Gdańsk (Poland) approved our study protocol (nr. NKBBN/521/2013). The research was performed in accordance with the Declaration of Helsinki. We obtained informed consent from all participants to use their CT images in this study and to publish the results.

### CFD

The Reynolds-Average Simulation (RAS) approach to turbulence was selected to perform the numerical simulation of the incompressible and dry airflow. Other approaches to flow modelling can also be considered, e.g. a direct solution of the Navier–Stokes equations or transitional turbulence models^9^. As for the RAS approach, a governing equation consists of the continuity equation

1 $$\begin{aligned} \nabla \cdot \bar{{\textbf{u}}} = 0, \end{aligned}$$

the Reynolds equation^23^

2 $$\begin{aligned} \frac{\partial \bar{{\textbf{u}}}}{\partial t} + \nabla \cdot \left( \bar{{\textbf{u}}} \bar{{\textbf{u}}} \right) = - \nabla \left( \tfrac{p}{\rho }+ \tfrac{2}{3} k \right) + \nabla \cdot \left( \left( \nu _t + \nu \right) 2 \, \bar{{\textbf{D}}} \right) , \end{aligned}$$

and two additional transport equations for the *k*-$\omega$ SST turbulence model^24^

3 $$\begin{aligned} \frac{\partial k}{\partial t} + \nabla \cdot \left( k \bar{{\textbf{u}}} \right) = 2 \nu _t \bar{{\textbf{D}}}^2 + \nabla \cdot \left( \left( \tfrac{\nu _t}{\sigma _{k3}} + \nu \right) \nabla k \right) - C_\mu k \omega , \end{aligned}$$

4 $$\begin{aligned} \begin{aligned} \frac{\partial \omega }{\partial t} + \nabla \cdot \left( \omega \bar{{\textbf{u}}} \right)&= \alpha _3 \frac{\omega }{k} 2 \nu _t \bar{{\textbf{D}}}^2 + \nabla \cdot \left( \left( \tfrac{\nu _t}{\sigma _{\omega 3}} + \nu \right) \nabla \omega \right) \\&-\beta _3 \omega ^2 + (1 - F_1) \frac{2}{\omega }\sigma _{\omega 3} \nabla k \cdot \nabla \omega , \end{aligned} \end{aligned}$$

with an additional equation for the eddy viscosity

5 $$\begin{aligned} \nu _t = a_1 k \, {\max }^{-1} \left( a_1 \omega , \sqrt{2 \bar{{\textbf{D}}}^2} F_2 \right) , \end{aligned}$$

where ${\textbf{u}}$ is the velocity vector, *p* is the pressure, $\rho$ – the constant density, $\nu$ – the kinematic viscosity coefficient, $\nu _t$ – the eddy viscosity, ${\textbf{D}}$ – the strain-rate tensor, *k* – the kinetic energy of velocity fluctuations and $\omega$ – the turbulence frequency. Moreover, the constants marked with the subscript ‘3’, such as $\sigma _{k3}$, $\sigma _{\omega 3}$, $\alpha _3$, $\beta _3$ are linear combinations of the constants from the component models. The additional constants are $a_1 = 0.31$, $C_\mu = 0.09$. The two blending functions are denoted here as $F_1$ and $F_2$.

The finite volume method discretises the governing equations^25,26^. Convection terms involved Gauss integration and were interpolated through cell-centered values utilising second-order accurate linear upwind interpolation. To maintain second-order accuracy for non-orthogonal meshes, an additional explicit non-orthogonal and limited correction was considered for the discretised diffusive terms. Velocity and pressure gradients utilised Gaussian integration and limited linear interpolation.

What is more, the fluxes also made use of linear interpolation. An implicit, three-level method (backward differencing) was used to discretise the time derivatives, and the transient system of equations was solved using the PISO algorithm^27^. The corrected pressure equation was solved utilising the GAMG solver with the combined diagonal-based incomplete Cholesky and Gauss-Seidel smoother. Smooth solvers using a Gauss-Seidel smoother were employed for the velocity fields *k* and $\omega$.

The study of the influence of the mesh on the solution was presented in our previous publication^28^. Moreover, the properties of the mesh and calculation times (Xeon 5120 2.2 GHz processor (13 out of 14 cores involved)) are given in Table 2. What is essential, the mesh size corresponds directly to computed tomography slice thickness and can be classified as Cartesian mesh (consists of mostly hexahedral elements).

**Figure 5:**
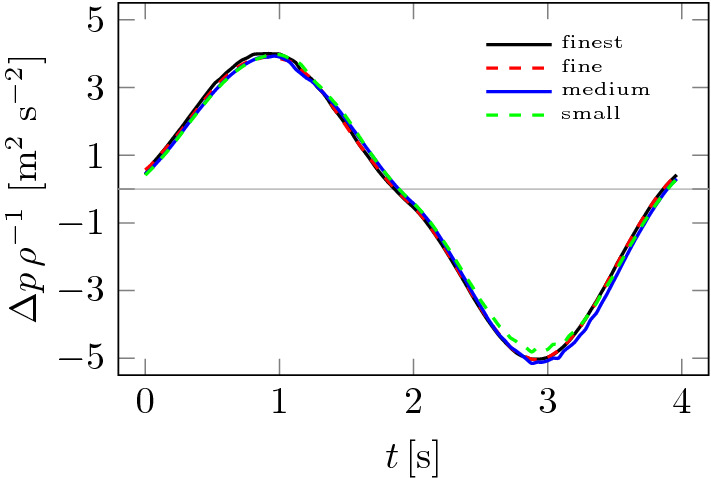
Influence of computational mesh on pressure drop $\Delta p$.

**Figure 6:**
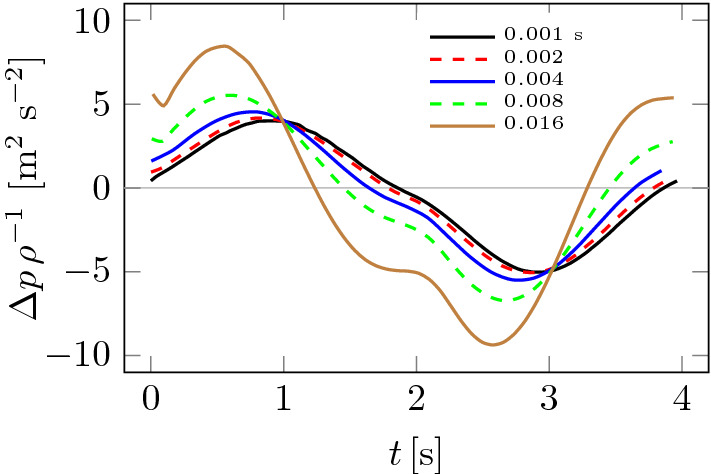
Influence of time step $\Delta t$ on pressure drop $\Delta p$.

A study of the effect of the computational mesh on the results of pressure drops is shown in Fig. 5. Four meshes were considered, the basic parameters of which are given in Table 1. It can be seen from Fig. 5 that the effect of the mesh on pressure drops, and therefore flow resistance NR, is small. This can be explained by the fact that pressure drops and therefore NR are quantities that depend mainly on the average inlet and outlet pressures. Furthermore, the effect of the choice of time step $\Delta t$ on pressure drops is shown in Fig. 6. In this case, the effect of the length of $\Delta t$ can be seen, especially for its large values. The computation time for the largest time step of $\Delta t = 0.016$ s was only 1.2 h. Moreover, the differences between the different pressure drop plots decrease as the time step decreases and are almost negligible for the case of $\Delta t = 0.002$ and $\Delta t = 0.001$. The latter case was adopted for the calculations.

**Table 1:** Mesh check statistics.

Mesh	Small	Medium	Fine	Finest
Nodes	3,759,502	5,855,577	8,252,115	10,152,642
Volumes	2,854,601	4,950,328	7,799,907	9,624,277
Computation [h]	4.9	7.9	13.3	17.5

A slightly more demanding analysis of the influence of the computational mesh on the results can be carried out using the so-called vorticity criterion *Q*, which can, for example, be used to compare velocity fields or flow configurations^28,29^. The *Q*-criterion is based on the velocity gradient $\nabla {\textbf{u}}$ tensor invariants^30^

6 $$\begin{aligned} {{\bar{Q}}} = \frac{1}{|V|} \int _V {\tfrac{1}{2} \left( \Vert \textbf{A}\Vert ^2 - \Vert {\textbf{D}}\Vert ^2 \right) {\mathrm{d}}V} \end{aligned}$$

where ${\textbf{D}}$ id symmetric and ${\textbf{A}}$ antisymmetric parts of the velocity gradient tensor $\nabla {\textbf{u}}$. Thus, the value of ${{\bar{Q}}}$ depends on the local derivatives of velocity, which can be influenced by computational meshes and the fluid motion’s local topology^31^.

**Figure 7:**
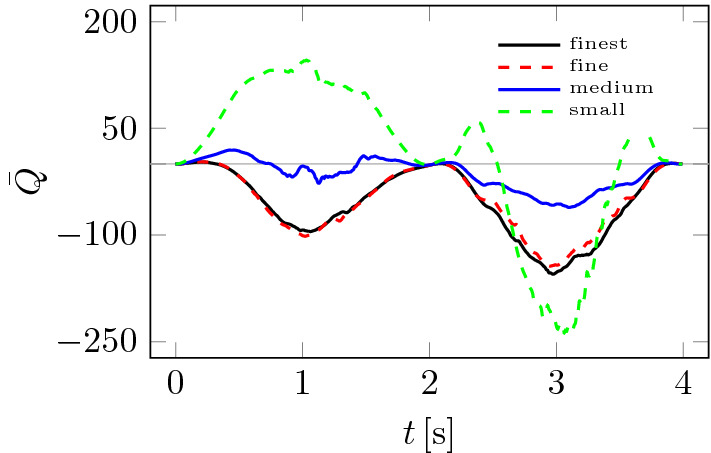
Influence of computational mesh on ${{\bar{Q}}}$.

**Figure 8:**
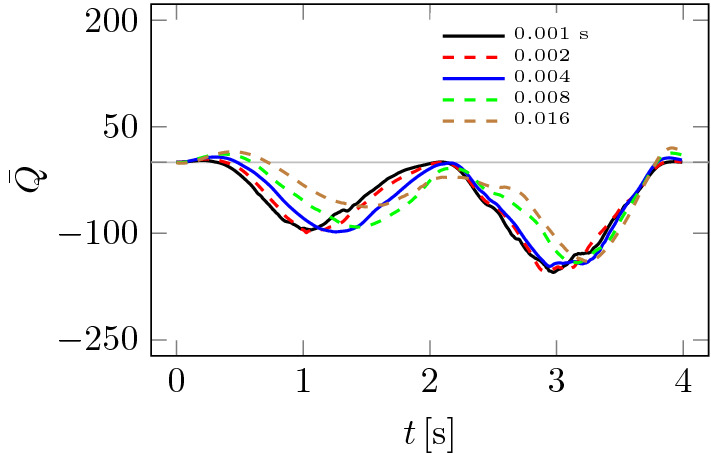
Influence of time step $\Delta t$ on ${{\bar{Q}}}$.

Figure 7 shows how significant the effect of mesh size on the ${{\bar{Q}}}$ value is compared to the pressure drops $\Delta p$ in Fig. 5. The effect ceases to be significant only between fine and finest meshes from Table 1. Figure 8 shows how the choice of time step $\Delta t$ changes the ${{\bar{Q}}}$ plots. As was the case in Fig. 7, the smaller the time step, the smaller the differences between solutions. Based on the analysis of Figs. 5, 6, 7 and 8, the finest mesh and the time step of $\Delta t = 0.001$ s were used for the calculations.

The inlet boundary condition localised at the larynx was specified through the volumetric flow rate corresponding to 5.1 litres per minute^9,28^. It was assumed that the typical breath took 4 seconds and that the exhalation and inhalation phases each lasted 2 seconds. The whole breathing cycle period was divided into 4000 fixed-time steps, corresponding to 0.001 seconds per step. Also, low turbulence intensity was assumed in order to calculate turbulence quantities *k* and $\omega$. The outlet surfaces were localised at the external nostrils where the assumed total pressure distribution equals atmospheric pressure. The remaining walls were regarded as no-slip walls with zero gradient pressure. Moreover, the scalable wall function modelled the flow in the region of the near walls.

Table 2 additionally shows time-averaged $\bar{{{\bar{Q}}}}$ values and average NR flow resistance. The averaged values are understood as the integral average over time *t* of an integral average over flow volume *V*, i.e.^28,29^

7 $$\begin{aligned} \bar{{{\bar{Q}}}} = \frac{1}{T} \int _0^{T} { {{\bar{Q}}}(t) \, {\mathrm{d}}t}. \end{aligned}$$

The lower subscript *e* indicates the exhalation phase and *i* – the inhalation phase.

**Table 2:** Patient data and results.

Patient	1	2
Condition	N	DSN
Sex	M	M
Age	38	33
Weight [kg]	84	89
Height [cm]	179	182
Volume $|V|$ [ml]	109.5	63.74
Nodes	10,152,642	6,519,472
Volumes	9,624,277	6,119,137
Computation [h]	17.5	9.1
$\text {NR}_e \,[\text {Pa s/ml}]$	0.0133	0.0204
$\text {NR}_i \,[\text {Pa s/ml}]$	0.0163	0.0924
$\bar{{{\bar{Q}}}}_e$	− 39.8	263.2
$\bar{{{\bar{Q}}}}_i$	− 73.5	− 5150.8

### Ethics approval and consent to participate

The protocol of this study was approved by the Regional Bioethics Committee at the Medical University of Gdansk, Poland (approval No. NKBBN/521/2013). Each patient gave written consent to use their CT images in this study.

## Data Availability

The datasets used and/or analysed during the current study available from the corresponding author on reasonable request.
